# Evaluation of iron accumulation during childhood cancer treatment

**DOI:** 10.3389/fped.2025.1619659

**Published:** 2026-01-05

**Authors:** Şule Çalışkan Kamış, Metin Çil, Begül Yağcı, Barbaros Şahin Karagün

**Affiliations:** Department of Pediatric Hematology and Oncology, Adana Faculty of Medicine, Adana City Education and Research Hospital, University of Health Sciences, Adana, Türkiye

**Keywords:** iron overload, T2* MRI, pediatric oncology, bone marrow transplantation, ferritin

## Abstract

**Background:**

Iron overload is a major concern in pediatric oncology, especially among patients receiving frequent blood transfusions. Although serum ferritin (SF) is widely used as a surrogate marker, T2* magnetic resonance imaging (MRI) of the liver and heart remains the gold standard for quantifying tissue iron deposition. This study evaluated the relationship between serum ferritin levels and T2* MRI findings in pediatric cancer patients, focusing on those with ferritin levels >1,000 µg/L.

**Methods:**

This retrospective study included patients aged 10–25 years diagnosed with malignancies and followed at the Pediatric Hematology and Oncology Clinic of Adana City Training and Research Hospital between June 2023 and December 2024. The upper age limit was chosen to include adolescent and young adult patients treated under pediatric oncology protocols. Serum ferritin and C-reactive protein (CRP) levels were measured during afebrile, infection-free periods. Elevated ferritin levels concurrent with high CRP were re-evaluated to exclude inflammation-related effects. Transfusion frequency and ferritin levels were recorded at 3, 6, and 12 months. Patients with ferritin >1,000 µg/L underwent cardiac and hepatic T2* MRI to assess organ-level iron burden and determine the need for chelation therapy.

**Results:**

Twenty-eight patients (median age: 14 years; 12 females, 16 males) were analyzed. The median baseline ferritin level was 32.5 µg/L. A significant correlation was found between transfusion number and ferritin >1,000 µg/L within the first 3 months (*p* = 0.029, *r* = 0.48) and for total annual transfusions (*p* = 0.001). Correlations at 3–6 months (*p* = 0.061, *r* = 0.42) and 6–12 months (*p* = 0.065, r = 0.39) were not statistically significant. Three patients (10.7%) had ferritin >1,000 µg/L—two with acute lymphoblastic leukemia and one with non-Hodgkin lymphoma. Among them, one underwent bone marrow transplantation, one died, and one had moderate hepatic but normal cardiac iron on T2* MRI.

**Conclusion:**

Iron overload is a preventable but clinically important complication in pediatric oncology. Combined monitoring with serum ferritin and T2* MRI enables early detection and timely management. Larger multicenter studies are needed to optimize screening intervals and chelation strategies in this high-risk group.

## Introduction

The diagnosis of childhood cancer is a life-altering event. With new treatment methods, the 5-year overall survival rate has improved to approximately 80% worldwide. Nevertheless, childhood cancers remain one of the leading causes of mortality among children aged 5–14 years (1, 2).

Supportive care procedures such as blood transfusions play a crucial role in pediatric cancer therapy; however, each unit of transfused red blood cells (RBCs) delivers approximately 200–250 mg of elemental iron.

When repeated erythrocyte transfusions are administered over the course of treatment, iron progressively accumulates in tissues, leading to transfusional iron overload (IO). IO has been recognized as a major cause of morbidity and mortality in pediatric hematology–oncology practice (3–5). Excessive iron deposition in vital organs, particularly the heart and liver, can result in life-threatening dysfunction and may adversely affect long-term survival (6–8).

Despite its clinical significance, studies investigating iron overload in children with malignancy remain limited, and the methods used to assess iron burden are heterogeneous (9, 10). Serum ferritin (SF) is widely used as a convenient, inexpensive, and non-invasive marker of body iron stores; however, SF levels are influenced by inflammation, infection, and liver injury, thus must be interpreted in conjunction with clinical and biochemical findings (11, 12).

**Figure 1:**
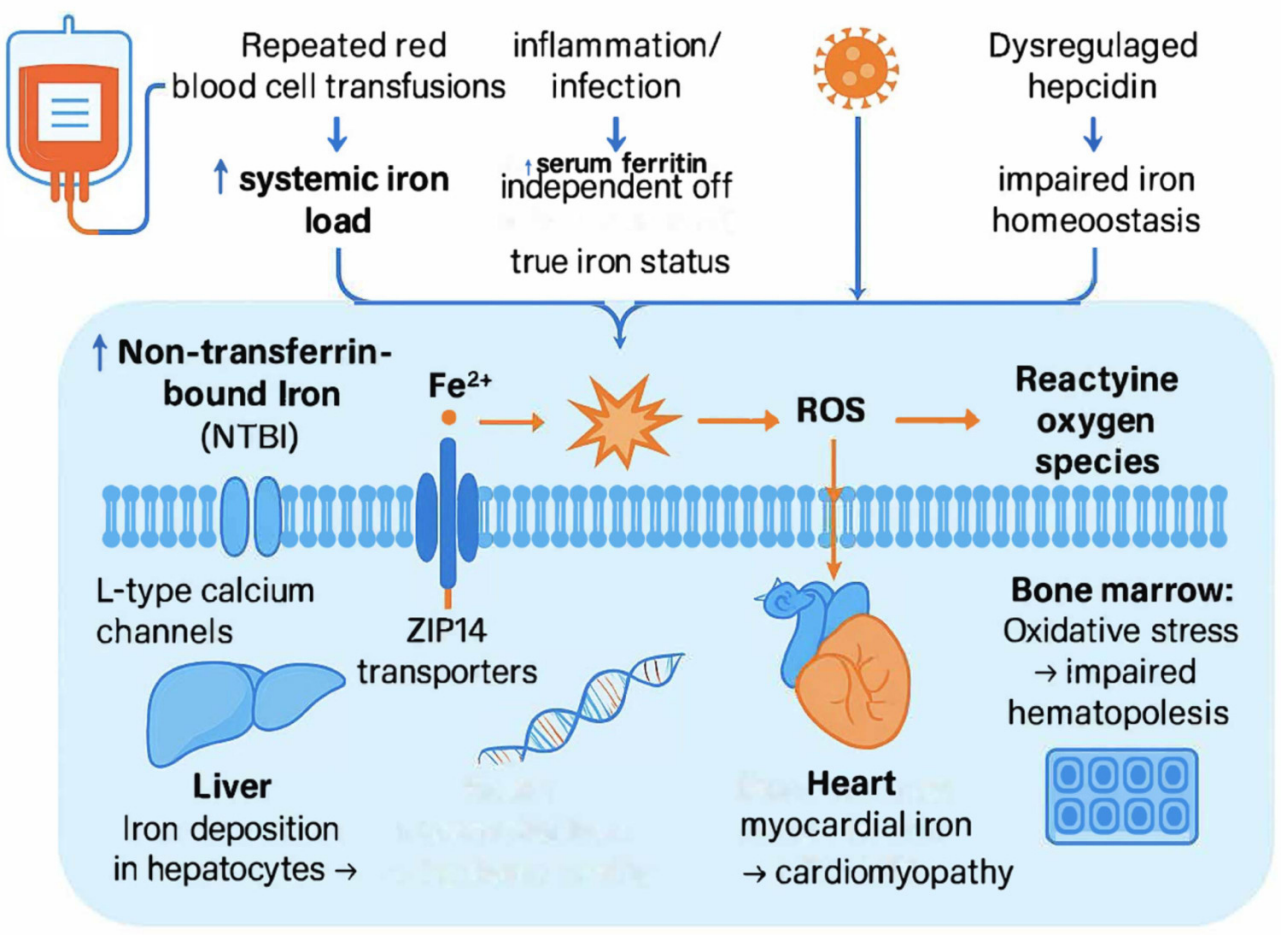
Mechanistic overview of transfusional iron overload in pediatric cancer patients. Repeated blood transfusions and inflammation increase systemic iron load and serum ferritin levels, leading to the accumulation of non–transferrin-bound iron (NTBI). Excess NTBI enters cells through L-type calcium channels and ZIP14 transporters, catalyzing the production of reactive oxygen species (ROS). These reactive molecules cause oxidative stress, resulting in hepatic and myocardial iron deposition and impaired hematopoiesis in the bone marrow. Early detection through combined serum ferritin and T2* MRI monitoring can prevent irreversible organ damage.

Magnetic resonance imaging (MRI) using T2* sequences provides a quantitative and highly sensitive assessment of tissue iron deposition, allowing simultaneous and non-invasive evaluation of hepatic and cardiac iron overload in pediatric cancer patients (13–15). Given the increasing survival and cumulative transfusion exposure in this population, integrating T2* MRI with serum ferritin monitoring may offer a more reliable approach for the early detection and management of iron overload. The mechanistic pathway of transfusional iron overload and its organ-specific effects in pediatric cancer patients are illustrated in Figure 1.

## Materials and methods

### Study design and setting

This retrospective observational study included patients aged 10–25 years who were diagnosed with childhood malignancies and followed at the Pediatric Hematology and Oncology Clinic of Adana City Training and Research Hospital (ACTRH) between June 1, 2023, and December 1, 2024.

The upper age limit of 25 years was selected to include adolescent and young adult (AYA) patients managed under pediatric oncology protocols at our institution, thereby ensuring uniformity in treatment approaches and data collection within a single clinical service.

### Laboratory assessments

SF levels were measured during afebrile, infection-free periods. C-reactive protein (CRP) was obtained concurrently to exclude inflammation-related ferritin elevation. If CRP was elevated, ferritin measurements were repeated because ferritin is an acute-phase reactant.

### Transfusion exposure and follow-up

During follow-up, the number of erythrocyte suspension (ES) transfusions and SF levels at 3, 6, and 12 months weredocumented. Patients with SF >1,000 µg/L underwent both hepatic and cardiac T2* MRI to assess organ-level iron accumulation and the potential need for chelation therapy.

### MRI acquisition

MRI examinations were performed on a 1.5T clinical scanner (Philips Ingenia MRI System, Eindhoven, Netherlands). Myocardial T2* sequences were acquired in a single short-axis view of the left ventricle using a breath-hold segmented multi-echo gradient-echo sequence. Liver T2* values were obtained from multiple parenchymal regions of interest (ROIs), avoiding vascular structures and bile ducts.

### Post-imaging management

Patients with confirmed hepatic or cardiac iron overload were evaluated for potential iron chelation therapy.

### Ethics

This study was approved by the ACTRH Clinical Research Ethics Committee (Decision No. 2555; May 11, 2023).

### Statistical analysis

The collected data were analyzed using SPSS (Statistical Package for the Social Sciences), version 26.0 (IBM Corp., Armonk, NY, USA).

Categorical variables were expressed as numbers and percentages, whereas continuous variables were summarized as mean ± standard deviation (SD) or median (range) when appropriate.

The Kolmogorov–Smirnov test was applied to assess the normality of continuous data. For comparisons between two independent groups, the Student's *t*-test was used for normally distributed variables, and the Mann–Whitney *U* test for non-normally distributed variables. Chi-square tests were performed for categorical data comparisons.

Correlation analysis was conducted to evaluate the relationships between quantitative variables. Pearson correlation coefficients were calculated for normally distributed data, and Spearman's rank correlation for non-normally distributed data.

A *p*-value ≤ 0.05 was considered statistically significant.

## Results

A total of 28 pediatric cancer patients were included in this study. Among them, 12 (42.9%) were female and 16 (57.1%) were male. The median age at diagnosis was 14 years (range: 10–17 years). The median serum ferritin (SF)level at diagnosis was 32.5 µg/L (range: 3–932), and the median C-reactive protein (CRP) level was 2 mg/L (range: 0–130).

The demographic and diagnostic characteristics of the study population are summarized in Table 1. The distribution of diagnoses was as follows: acute lymphoblastic leukemia (ALL) in 3 patients (10.7%), Hodgkin lymphoma (HL) in 6 (21.4%), non-Hodgkin lymphoma (NHL) in 3 (10.7%), osteosarcoma in 3 (10.7%), rhabdomyosarcoma (RMS) in 1 (3.6%), alveolar soft part sarcoma in 2 (7.1%), ependymoma in 2 (7.1%), choroid plexus carcinoma in 1 (3.6%), mature cystic teratoma in 2 (7.1%), chronic myeloid leukemia (CML) in 1 (3.6%), fibroadenoma in 2 (7.1%), Leydig cell tumor in 1 (3.6%), and germ cell tumor in 1 (3.6%).

**Table 1:** Characteristics and diagnostic distribution of pediatric cancer patients included in the study.

Characteristic	Value
Total number of patients	28
Sex distribution	Female: 12 (42.9%)
	Male: 16 (57.1%)
Median age at diagnosis (years)	14 (range: 10–17)
Median ferritin level (µg/L)	32.5 (range: 3–932)
Median CRP level (mg/L)	2 (range: 0–130)
Diagnoses	
Acute Lymphoblastic Leukemia (ALL)	3 (10.7%)
Hodgkin Lymphoma (HL)	6 (21.4%)
Non-Hodgkin Lymphoma (NHL)	3 (10.7%)
Osteosarcoma	3 (10.7%)
Rhabdomyosarcoma (RMS)	1 (3.6%)
Alveolar Soft Part Sarcoma	2 (7.1%)
Ependymoma	2 (7.1%)
Choroid Plexus Carcinoma	1 (3.6%)
Mature Cystic Teratoma	2 (7.1%)
Chronic Myeloid Leukemia (CML)	1 (3.6%)
Fibroadenoma	2 (7.1%)
Leydig Cell Tumor	1 (3.6%)
Germ Cell Tumor	1 (3.6%)

Statistically significant correlations were identified between the number of blood transfusions and serum ferritin levels exceeding 1,000 µg/L across various time intervals: within the first 3 months (*p* = 0.029, r = 0.48) and for the total annual number of transfusions (*p* = 0.001).

**Figure 2:**
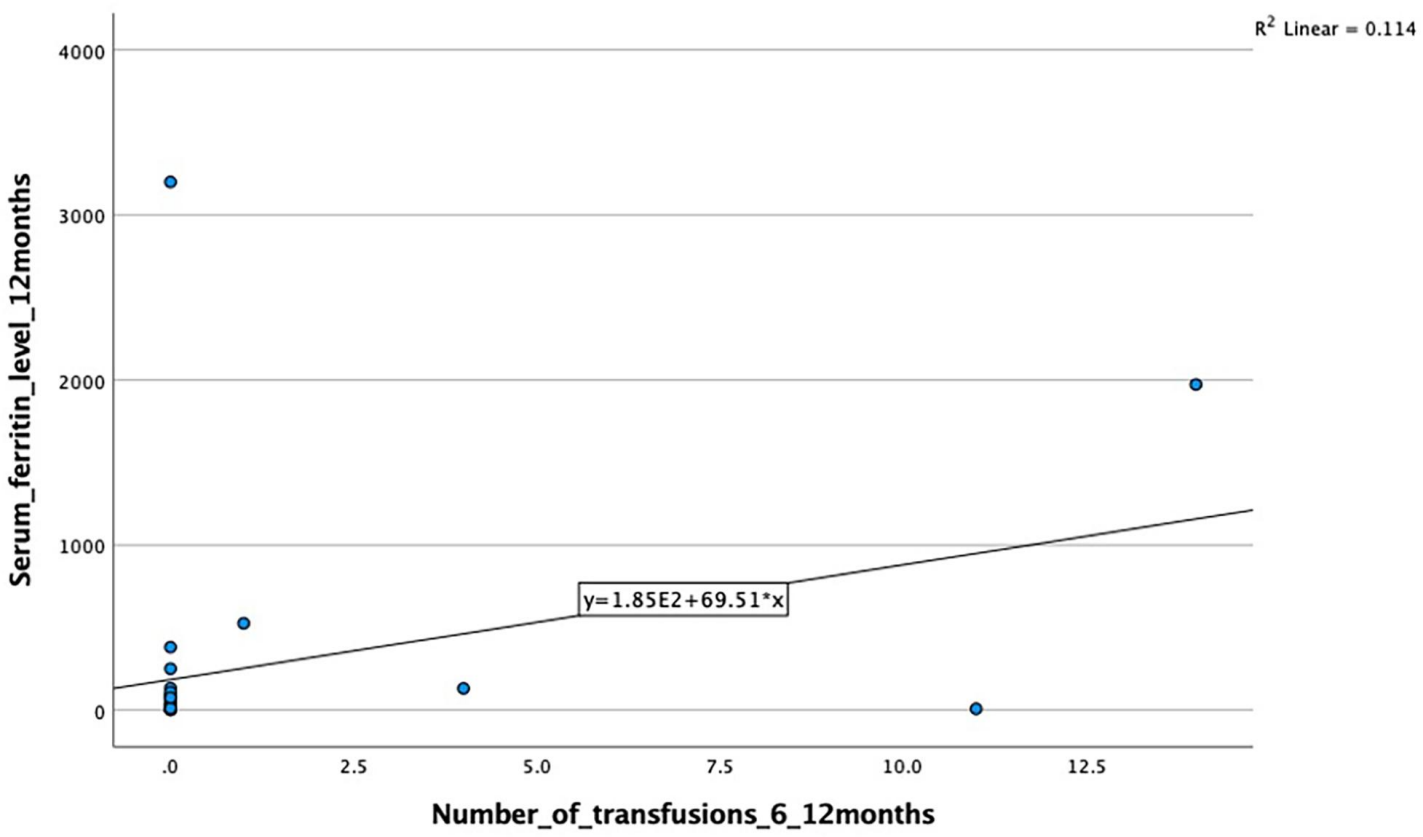
Correlation between transfusion number (6–12 months) and 12-month serum ferritin levels. A moderate positive correlation was observed (*r* = 0.39), but it did not reach statistical significance (*p* = 0.065).

A moderate positive correlation was also observed between the number of transfusions and serum ferritin levels at 12 months, although it did not reach statistical significance (*r* = 0.39, *p* = 0.065) (Figure 2).

However, the associations at 3–6 months (*p* = 0.061, *r* = 0.42) and 6–12 months (*p* = 0.065, *r* = 0.39) were not statistically significant and should be interpreted as non-significant trends attributable to the limited sample size.

Additionally, a significant correlation was observed between the annual number of transfusions and ferritin levels at 12 months using Spearman's correlation test (*p* = 0.002, *r* = 0.569), as well as between patient diagnosis and ferritin levels exceeding 1,000 µg/L (*p* = 0.03, *r* = 0.410).

Among the three patients with ferritin levels >1,000 µg/L, two were diagnosed with ALL and one with NHL. Detailed clinical characteristics, ferritin trends, and T2* MRI findings of these patients are presented in Table 2.

**Table 2:** Clinical characteristics and iron overload Status of patients with ferritin levels exceeding 1,000 µg/L.

Patient no	Diagnosis	Status	Myocardial T2* (ms)	Hepatic T2* (ms)	Ferritin level (µg/L)	Chelation therapy	Iron deposition/notes
1	ALL	Deceased	–	–	>1,000	No	Undetermined
2	ALL	Underwent bone marrow transplantation	35	4.72	1,000 → 384 (at 12 months)	No	Moderate (liver); cardiac value normal. Ferritin likely transplant-related.
3	NHL	T2* cardiac and hepatic MRI performed	134	7.3	>1,000	No	Moderate (liver); cardiac value normal.

Reference ranges:

Myocardial T2*: >20 ms (normal).

Hepatic T2*: >11.4 ms (normal), 3.8–11.4 ms (moderate).

For the patient who underwent MRI, the myocardial T2* value measured in the interventricular septum was 134 ms, which is within the normal range (>20 ms). In contrast, the hepatic T2* value was 7.3 ms, indicating moderate iron accumulation in the liver parenchyma (>11.4 ms: normal; 3.8–11.4 ms: moderate).

Post-transplant T2* MRI scans were also performed in the patient who underwent bone marrow transplantation (BMT). The cardiac T2* value was 35 ms (normal >20 ms), while the hepatic T2* value was 4.72 ms, again indicating moderate hepatic iron deposition.

**Figure 3:**
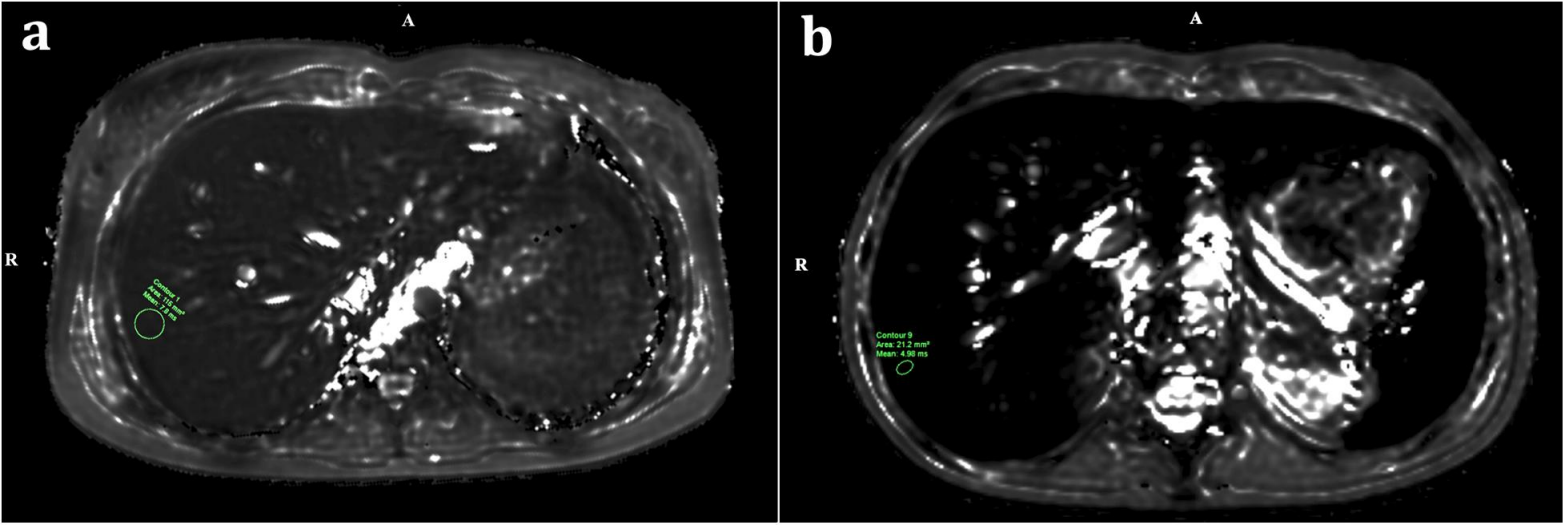
Representative hepatic T2* MRI findings in pediatric oncology patients with serum ferritin >1,000 µg/L. **(a)** Axial hepatic T2*-weighted MRI image from a patient with acute lymphoblastic leukemia (ALL) demonstrates moderate hepatic iron deposition, with a mean hepatic T2* value of 7.3 ms (normal >11.4 ms). **(b)** Axial hepatic T2*-weighted MRI image from another patient with elevated ferritin after bone marrow transplantation (BMT) shows moderate hepatic iron overload, with a mean hepatic T2* value of 4.72 ms. These images illustrate that T2* MRI provides sensitive, quantitative assessment of hepatic iron burden, complementing serum ferritin monitoring in pediatric patients receiving repeated transfusions.

Despite moderate hepatic iron overload, the ferritin level, which had exceeded 1,000 µg/L at 3 months, decreased to 384 µg/L by 12 months without chelation therapy. In the patient who underwent BMT and had ferritin >1,000 µg/L, the elevated ferritin level was considered transplant-related rather than secondary to transfusional iron accumulation. Representative hepatic T2* MRI images demonstrating iron accumulation are shown in Figure 3.

## Discussion

Iron overload (IO) remains a significant clinical concern in pediatric oncology, particularly in patients undergoing intensive transfusion support and bone marrow transplantation (BMT). In our cohort, although only three patients (10.7%) demonstrated ferritin levels exceeding 1,000 µg/L, T2* MRI revealed moderate hepatic iron accumulation in two of them. These results indicate that even a relatively small number of transfusions may lead to early organ-level iron deposition, underscoring the need for regular and combined monitoring with serum ferritin and T2* MRI.

The small sample size and descriptive nature of our findings represent the major limitation of this study; therefore, the results should be interpreted as preliminary observations requiring validation in larger cohorts. Nevertheless, the presence of moderate hepatic iron accumulation in patients with high ferritin values supports previous evidence that serum ferritin alone may not accurately reflect tissue iron burden, particularly in patients with fluctuating inflammatory status.

Iron overload following BMT is a well-recognized complication that can adversely affect survival outcomes. Our findings align with those of Halonen et al., who reported that iron overload was detected in approximately 14% of children treated for acute lymphoblastic leukemia (16). Elevated iron levels exert toxic effects through the formation of reactive oxygen species (ROS), which can damage DNA, lipids, and proteins, leading to oxidative stress and organ dysfunction (17).

Preclinical studies have shown that iron toxicity alters the expression of genes involved in hematopoiesis and cytokine regulation, disrupting the bone marrow microenvironment. These effects may be partially reversed by iron chelation therapy, such as with deferasirox, which improves oxidative balance and stem cell function (18). Moreover, studies in animal models have demonstrated delayed hematopoietic recovery after transplantation due to excess iron, accompanied by reduced erythropoietin and thrombopoietin expression (19). Such findings highlight the biological plausibility of iron-induced hematopoietic suppression observed in pediatric patients receiving multiple transfusions.

In the present study, one patient's ferritin level declined spontaneously from >1,000 µg/L to 384 µg/L without chelation therapy. This may be explained by the cessation of transfusions, improved inflammatory status, or increased iron utilization during growth and recovery, suggesting that ferritin fluctuations should always be interpreted in the clinical context rather than as an isolated biochemical indicator.

Furthermore, Pullarkat et al. demonstrated that iron overload adversely affects outcomes after allogeneic hematopoietic cell transplantation, emphasizing that high pre-transplant ferritin levels are associated with increased mortality and transplant-related complications (20). This supports our observation that elevated ferritin in post-transplant patients may reflect a transient inflammatory or transplant-related process, yet persistent hyperferritinemia should prompt evaluation for true tissue iron overload by T2* MRI.

Overall, excessive iron accumulation represents a preventable yet underestimated complication in pediatric oncology and transplantation. Our findings emphasize the importance of early identification of high-risk patients, the integration of T2* MRI into follow-up protocols, and the consideration of timely chelation therapy to protect organ function and long-term survival.

Future multicenter studies with larger sample sizes and longitudinal follow-up are warranted to determine optimal monitoring intervals, ferritin thresholds, and treatment algorithms for managing iron overload in pediatric cancer survivors.

## Conclusion

In conclusion, the interaction between cancer therapy, transfusion practices, and iron metabolism underscores the need for an integrated approach to iron management in pediatric oncology patients. Our findings highlight that persistent monitoring of serum ferritin and, when indicated, confirmatory T2* MRI assessments are essential—particularly for patients undergoing bone marrow transplantation (BMT)—to prevent the potentially life-threatening consequences of iron overload.

Iron toxicity exerts detrimental effects on hematopoiesis and organ function, which may compromise long-term survival. Therefore, early recognition and intervention through optimized transfusion protocols and timely initiation of iron chelation therapy should be integral to post-treatment follow-up in pediatric cancer survivors.

Future studies with larger, multicenter cohorts are needed to establish standardized thresholds for intervention and to refine chelation regimens, thereby improving long-term outcomes and quality of life in this vulnerable patient population.

## Data Availability

The raw data supporting the conclusions of this article will be made available by the authors, without undue reservation.
